# Changes of Ovarian microRNA Profile in Long-Living Ames Dwarf Mice during Aging

**DOI:** 10.1371/journal.pone.0169213

**Published:** 2017-01-03

**Authors:** Augusto Schneider, Scot J. Matkovich, Berta Victoria, Lina Spinel, Andrzej Bartke, Pawel Golusinski, Michal M. Masternak

**Affiliations:** 1 Faculdade de Nutrição, Universidade Federal de Pelotas, Pelotas, RS, Brazil; 2 College of Medicine, Burnett School of Biomedical Sciences, University of Central Florida, Orlando, FL, United States of America; 3 Center for Pharmacogenomics, Department of Medicine, Washington University School of Medicine, St. Louis, MO, United States of America; 4 Departments of Internal Medicine and Physiology, Southern Illinois University School of Medicine, Springfield, IL, United States of America; 5 Department of Biology and Environmental Studies, Poznan University of Medical Sciences, Poznan, Poland; 6 Department of Head and Neck Surgery, The Greater Poland Cancer Centre, Poznan, Poland; Kunming University of Science and Technology, CHINA

## Abstract

The Ames dwarf (df/df) mice have extended longevity and can preserve the ovarian reserve longer than Normal (N) mice. Based on this, the aim of our study was to evaluate the ovarian microRNA (miRNA) profile in young and aged df/df and N mice. Ovarian tissue was collected at 5–6 months and at 21–22 months of age for miRNA sequencing. We detected a total of 404 miRNAs in the ovarian samples, from which the abundance of 22 and 33 miRNAs changed with age in N and df/df mice, respectively. Of these, only three miRNAs were commonly regulated with age between N and df/df mice, indicating a very divergent miRNA profile between genotypes. We also detected that 46 miRNAs were regulated between N and df/df mice, of which 23 were regulated exclusively in young mice, 12 exclusively in old mice and 12 commonly regulated at young and old ages. Many genes likely to be targeted by these miRNAs are involved in the FoxO, mTOR, PI3k/Akt and insulin signaling pathways. These results suggest that the aging process has a differential impact on the ovarian miRNA profile in df/df mice, and suggest that these miRNAs can be central players in the maintenance of a younger ovarian phenotype.

## Introduction

Regulation of gene expression plays a key role in follicle development and aging within the ovary [1]. Some key cellular processes, including mRNA transcription and stability, are regulated by small non-coding RNAs (sncRNAs), which includes short sequences of about 20 nucleotides known as microRNAs (miRNAs) [2]. When miRNAs are exported to the cytoplasm, they modulate gene expression post-transcriptionally by interacting with the Argonaute proteins, forming an RNA Induced Silencing Complex (RISC) that binds to the 3’ untranslated region (UTR) and cleaves target mRNAs [3]. In addition to that, miRNAs can be secreted to the extracellular space [4] and have a role in intercellular communication in a hormone-like pattern between different cell types [5]. Therefore, miRNAs play an essential role in ovarian follicle development [6, 7], although little is known about the how miRNAs are regulated during the ovarian aging process [8, 9].

Oocytes enclosed in primordial follicles constitute the ovarian reserve and remain dormant until activated to grow during the female reproductive life [10]. During this period there is a progressive decline and depletion of the ovarian primordial follicle reserve culminating in the menopause [11]. In the female mouse the size of the ovarian reserve reduces 10 fold from six to 18 months of age [12]. Some miRNAs are known to be involved in premature ovarian failure in the rat [13] and were observed to be differentially regulated in the ovaries of ageing women [8, 9]. This evidence suggests that there is an association between miRNA expression and regulation of ovarian aging. However, no specific studies have been performed using unbiased, transcriptome-wide methods to determine miRNA changes during normal aging in mice with parallel comparison of these alterations in long-living animals such as the Ames dwarf (Prop-1^df^; df/df) mice.

The df/df mice have a defective Prop1 (Prophet of Pit1) gene, impairing anterior pituitary gland development, and resulting in deficient secretion of growth hormone (GH), thyroid-stimulating hormone (TSH) and prolactin [14]. These mice are characterized by severely reduced circulating IGF-I and adult body size [14]. The df/df mice are considered excellent models for the study of aging, since they can live 35–75% longer than their normal littermates [14], and share several characteristics with GH receptor gene disrupted mice and mice subjected to caloric restriction [15]. Although female df/df mice have normal ovarian cyclicity and can maintain pregnancy under hormonal stimulation, they have delayed puberty [16]. We previously reported that Forkhead Box O3a (Foxo3a) phosphorylation, one of the main pathways implicated in irreversible primordial follicle activation [17, 18], is reduced in oocytes enclosed in primordial follicles from df/df mice, preventing primordial follicle activation [19, 20]. As a result df/df mice have a delayed ovarian aging and an increased number of primordial follicles compared to old Normal (N) mice [19, 20]. Activation of primordial follicle growth is also impaired in GH receptor disrupted mice [21] and mice subjected to caloric restriction [22], also culminating in delayed ovarian aging and a greater number of primordial follicles in the quiescent stage. On the other hand, transgenic mice overexpressing GH have increased activation of Foxo3a and accelerated loss of primordial follicles [19], evidencing the role of GH/IGF-I in this process.

microRNAs are well known for their roles in cell growth and proliferation, regulating pathways which are also essential to cancer development [23], and there is an extensive characterization of the role of microRNAs in the etiology of ovarian cancer [24]. Epithelial ovarian cancers represent nearly 90% of all ovarian cancers and develop mainly in postmenopausal women [25]. In parallel with an increase in life expectancy, the rate of female reproductive cancer incidence has nearly doubled in the past 30 years [26]. The current hypothesis, supported by epidemiological and experimental evidence, suggests that repetitive ovulation and wounding of the ovarian surface during reproductive life can increase the risk of epithelial ovarian cancers [25], and most ovarian cancers usually develop immediately after menopause [27]. Therefore, a better understanding of ovarian aging and its links to known ovarian cancer markers can benefit healthy aging for women, since the current evidence supports that miRNAs may play a key role in both processes.

It was previously demonstrated, using the same mice analyzed in the present study, that df/df mice have a characteristic circulating miRNA signature associated with aging [28]. However, very little is known about the miRNA profile associated with ovarian aging, since most mouse models for ovarian aging (knockouts for PTEN and FoxO3 for example) have complete ovarian failure early after puberty [17, 29]. df/df mice can maintain the ovarian reserve for a longer period than N mice [19, 20], which allows for comparison of expression profiles at different stages of life, making it a very valuable model of ovarian aging. Therefore, the aim of the current work is to evaluate the ovarian miRNA profile in young and aged df/df and N mice in order to establish miRNAs potentially involved in ovarian aging.

## Materials and Methods

Ames dwarf mice (df/df; n = 10) Normal control littermates (N; n = 10) (all females) were bred and maintained under temperature- and light-controlled conditions (22 ± 2°C, 12 hour light/12 hour dark cycle) [30]. Mice were subjected to overnight fasting, and isofluorane was used for anesthesia and after blood collection by cardiac puncture mice were euthanized by performing cervical dislocation. Mice were dissected and the pair of ovaries was collected and stored at -80°C. The ovarian collection was performed at 5–6 months of age (young; 5 N and 5 df/df mice) and at 21–22 months of age (old; 5 N and 5 df/df mice). All animal procedures employed in our presented work were approved by and performed in accordance to the guidelines from the Laboratory Animal Care and Use Committee (LACUC) at the Southern Illinois University School of Medicine (Springfield, IL).

The pair of ovaries was removed from the -80°C and homogenized with Qiazol (Qiagen, Valencia, CA, USA) using 0.5 mm zirconium oxide beads in the Bullet Blender 24 (Next Advance, Averill Park, NY, USA). Total RNA was extracted using a commercial column purification system (miRNeasy Mini Kit, Qiagen) and on-column DNase treatment (RNase-free DNase Set, Qiagen) following manufacturer's instructions. The quantity and quality of RNA samples was determined using BioAnalyzer and RNA Nano Lab Chip Kit (Agilent Technologies, Santa Clara, CA, USA) and only samples with RNA Integrity Number higher than 7.0 were selected for further analysis (n = 20).

MicroRNA libraries were prepared using the TruSeq Small RNA Sample Prep Kit (Illumina Inc., San Diego, CA, USA) following the manufacturer's instructions according to Matkovich, Hu [31]. Briefly, small RNAs from 1 μg of total RNA were ligated with 3′ and 5′ adapters, followed by reverse transcription to produce single stranded cDNAs. Samples were amplified by PCR in 14 cycles using indexes to allow all 20 individual libraries to be processed in a single flowcell lane during sequencing. The amplified libraries were size-selected and purified in a 6% acrilamide gel, combined in a single microtube and submitted to sequencing on a HiSeq 2500 instrument (Illumina Inc.) at the Washington University Genomic Technology Access Center (GTAC). Alignment and quantification of miRNA libraries was performed using sRNAtoolbox as described before [32]. Briefly, sequences in the fasta format were adaptor trimmed, the sequences were aligned to the mouse genome (mm10) and known miRNA sequences confirmed by miRBase, and the output read count was expressed as normalized reads per million (rpm).

Statistical analyses of differentially expressed miRNAs was performed using EdgeR [33] between each pair of groups and miRNAs with a FDR<0.02 and FC>1.5 were considered as up or down-regulated. The unsupervised hierarchical clustering heatmap from the variance stabilization transformed data of the top 50 most expressed miRNAs ordered by average count was performed using the software R (3.2.2) and the Bioconductor package DESeq (1.2.0) [34]. miRNA gene families were identified using miRBase [35]. The mirPath tool (version 3.0) was used to predict target genes of the differentially regulated miRNAs using the microT-CDS v.5.0 database [36] and for retrieving gene ontology (GO) terms (biological processes) and KEGG molecular pathways [37, 38], considering a corrected P value lower than 0.05 as significant for pathway enrichment.

All miRNA-Seq data are available at the Gene Expression Omnibus (GEO) at NCBI under accession number GSE87518.

## Results

A total of 404 miRNAs were detected in the ovaries of N and df/df mice (present in at least 50% of the samples with more than 5 reads per million). On average 8,648,485 raw reads per sample were obtained, and from these 6,603,534 adaptor cleaned unique reads were used per sample, and 43.9% of those were aligned to the mouse genome. The overall percentage of the aligned reads mapped to different categories of sncRNA was: 57.9% for miRNAs, 28.7% for repeated sequences, 4,3% for snRNA, 4.2% for rRNA and 0.1% for snoRNA. About 10% of the detected miRNAs had more than 5,000 rpm, 13% between 1,000 and 5,000 rpm, 30% between 100 and 1,000 rpm, 37% between 10 and 100 rpm and 8% between 5 and 10 rpm.

**Fig 1:**
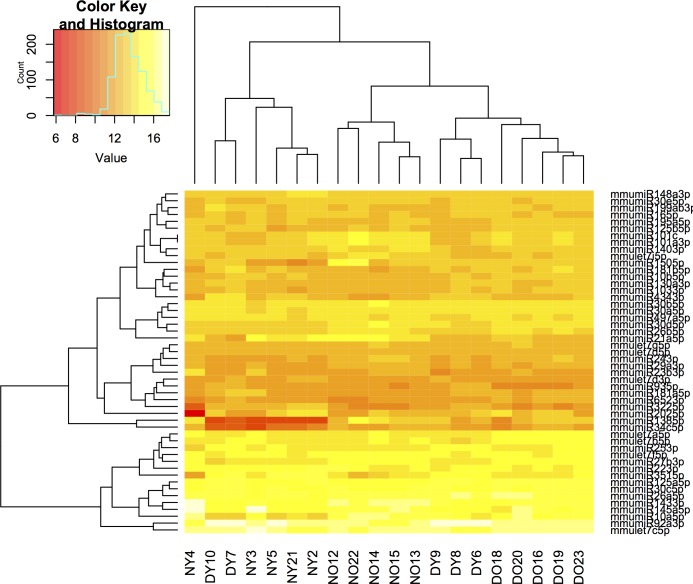
Unsupervised hierarchical clustering ordered by the adjusted level of miRNA expression for the top 50 most expressed miRNAs in ovaries of df/df (n = 10; Young–DY and Old–DO) and N mice (n = 10; Young–NY and Old–NO).

**Fig 2:**
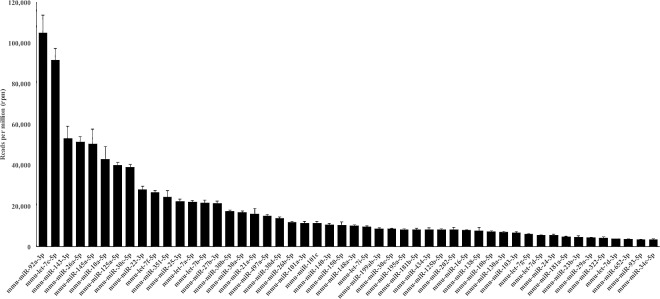
Abundance of the top 50 most expressed miRNAs in ovarian samples. The values are an average reads per million (rpm) from Ames dwarf (df/df) and Normal (N) mice at 6 and 22 months of age.

The 50 highest expressed miRNAs in the mice ovaries are represented in a heatmap in Fig 1 and for miRNA expression levels in Fig 2. The top five most expressed miRNAs in the ovary (all samples combined) were mmu-miR-92a-3p (miR-25 family), mmu-let-7c-5p (let-7 family), mmu-miR-143-3p (miR-143 family), mmu-miR-26a-5p (miR-26 family) and mmu-miR-145a-5p (miR-145 family). Pathway enrichment analysis indicated that these top five miRNAs target genes that are likely to be involved in important ovarian signaling pathways (FoxO, MAPK, AMPK, mTOR, PI3K/Akt, TGF-β, Estrogen and GnRH; for further details see S1 Table) and biological processes (S1 Table).

**Fig 3:**
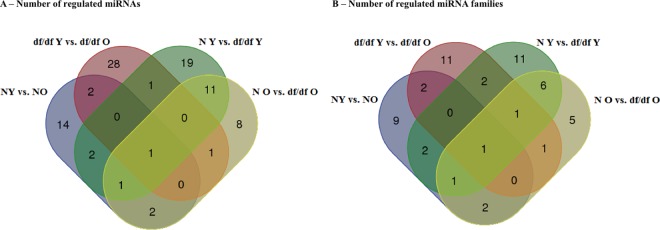
Venn diagram indicating individual miRNAs (A) and miRNAs gene families (B), based on miRBase classification of highly similar miRNAs, that were significantly regulated between Ames dwarf (df/df) mice with aging, and between young (6 months old) and aged (22 months old) df/df and Normal (N) mice.

There were 11 down-regulated and 11 up-regulated miRNAs in N mice with age (N young vs. N old) (FDR<0.02). In df/df mice, 31 miRNAs were down-regulated with age, while two were up-regulated (FDR<0.02; Table 1). When comparing miRNA expression between df/df and N mice, 12 miRNAs were commonly down-regulated at both young and old ages (five down- and seven up-regulated; FDR<0.02; Table 2), 23 were regulated only in young mice (13 down- and 10 up-regulated; FDR<0.02; Table 2) and 12 miRNAs were regulated exclusively in old mice (4 down- and 8 up-regulated; FDR<0.05; Table 2). A Venn diagram with the mutually regulated miRNAs between different categories is presented in Fig 3A and the number of regulated miRNA families is represented in Fig 3B.

**Table 1:** MicroRNAs differentially expressed during aging in Normal and Ames dwarf mice.

miRNA name	miRNA gene family	FC^1^	RPM^2^	PValue^3^	FDR^3^	miRNA name	miRNA gene family	FC^1^	RPM^2^	PValue^3^	FDR^3^
Normal mice						Ames dwarf mice					
**Commonly down-regulated**						**Commonly down-regulated**					
mmu-miR-217-5p	miR-217	23.83	10.53	9.36E-04	1.75E-02	mmu-miR-217-5p	miR-217	4.93	10.53	1.05E-04	2.50E-03
mmu-miR-410-3p	miR-154	2.45	69.50	6.12E-04	1.37E-02	mmu-miR-410-3p	miR-154	3.65	69.50	7.48E-07	5.00E-05
mmu-miR-673-5p	miR-673	2.35	42.02	2.13E-04	7.16E-03	mmu-miR-673-5p	miR-673	3.00	42.02	9.89E-07	5.00E-05
**Down-regulated only in Normal mice**						**Down-regulated only in Ames dwarf mice**					
mmu-miR-6538	-	19.43	92.01	5.83E-05	2.94E-03	mmu-miR-298-5p	miR-298	12.01	40.51	1.22E-09	4.94E-07
mmu-miR-450b-5p	miR-450	7.37	8.46	5.86E-04	1.37E-02	mmu-miR-296-3p	miR-296	7.60	18.12	9.88E-07	5.00E-05
mmu-miR-370-3p	miR-370	6.34	9.62	1.00E-05	8.08E-04	mmu-miR-296-5p	miR-296	6.37	88.46	4.88E-08	8.97E-06
mmu-miR-5126	-	5.85	34.48	2.82E-04	8.46E-03	mmu-miR-130b-3p	miR-130	5.55	101.52	6.66E-08	8.97E-06
mmu-miR-503-5p	miR-503	4.08	52.67	2.93E-04	8.46E-03	mmu-miR-130b-5p	miR-130	5.44	20.57	1.57E-06	7.04E-05
mmu-miR-351-3p	miR-351	3.24	98.17	1.57E-04	5.78E-03	mmu-miR-301b-3p	miR-130	4.94	69.68	6.49E-06	2.62E-04
mmu-miR-485-3p	miR-485	3.06	21.31	3.42E-04	9.21E-03	mmu-miR-486b-3p	miR-486	4.61	8.83	4.85E-04	7.75E-03
mmu-miR-3102-3p	miR-3102	2.25	104.61	9.01E-04	1.75E-02	mmu-miR-486a-3p	miR-486	4.61	8.83	4.82E-04	7.75E-03
						mmu-miR-381-3p	miR-154	4.46	9.14	9.67E-05	2.44E-03
**Up-regulated only in Normal mice**						mmu-miR-17-5p	miR-17	4.16	552.57	2.03E-07	2.05E-05
mmu-miR-215-5p	miR-192	21.95	191.47	3.04E-06	3.07E-04	mmu-miR-20a-5p	miR-17	3.91	342.08	9.89E-07	5.00E-05
mmu-miR-142a-3p	miR-142	7.79	586.99	1.04E-06	1.40E-04	mmu-miR-323-3p	miR-154	3.17	19.08	2.57E-04	5.42E-03
mmu-miR-205-5p	miR-205	6.47	541.62	2.82E-05	1.63E-03	mmu-miR-299b-5p	miR-299	3.03	33.57	7.97E-05	2.15E-03
mmu-miR-187-3p	miR-187	6.13	147.95	1.74E-05	1.17E-03	mmu-miR-299a-5p	miR-299	3.03	33.57	7.98E-05	2.15E-03
mmu-miR-146a-5p	miR-146	4.80	1783.78	4.45E-07	8.98E-05	mmu-miR-409-3p	miR-154	3.00	66.85	1.63E-05	5.50E-04
mmu-miR-142a-5p	miR-142	4.43	258.92	9.59E-05	4.31E-03	mmu-miR-494-3p	miR-154	2.87	158.07	4.99E-04	7.75E-03
mmu-miR-150-5p	miR-150	4.32	9575.94	2.43E-07	8.98E-05	mmu-miR-341-3p	miR-341	2.78	114.55	2.12E-05	6.59E-04
mmu-miR-146b-5p	miR-146	3.06	992.32	8.33E-04	1.75E-02	mmu-miR-329-3p	miR-329	2.77	15.86	9.24E-04	1.20E-02
mmu-miR-223-5p	miR-223	2.38	36.30	9.51E-04	1.75E-02	mmu-miR-667-3p	miR-667	2.70	144.89	2.95E-04	5.67E-03
mmu-miR-29c-5p	miR-29	2.32	114.74	1.16E-04	4.67E-03	mmu-miR-376a-5p	miR-368	2.47	46.24	2.68E-04	5.42E-03
mmu-miR-29c-3p	miR-29	2.29	1613.93	3.86E-04	9.76E-03	mmu-miR-434-3p	miR-434	2.34	7570.42	1.62E-05	5.50E-04
						mmu-miR-434-5p	miR-434	2.18	42.45	8.37E-04	1.18E-02
						mmu-miR-93-3p	miR-17	2.16	32.23	8.80E-04	1.18E-02
						mmu-miR-17-3p	miR-17	2.15	82.36	8.67E-04	1.18E-02
						mmu-miR-181c-5p	miR-181	2.12	212.90	4.11E-04	7.22E-03
						mmu-miR-379-3p	miR-379	2.10	73.53	8.19E-04	1.18E-02
						mmu-miR-181c-3p	miR-181	2.07	1108.31	3.31E-04	6.08E-03
						mmu-miR-93-5p	miR-17	1.99	3218.94	1.35E-04	3.03E-03
						**Up-regulated only in Ames dwarf mice**					
						mmu-miR-592-5p	miR-592	3.97	16.28	1.02E-03	1.28E-02
						mmu-miR-10b-3p	miR-10	1.93	70.41	1.38E-03	1.69E-02

^1^Fold change between groups

^2^Average reads per million (RPM) for each miRNA for the groups being compared

^3^P Value and False discovery rate. miRNAs with FDR<0.05 were considered as differentially expressed between groups

**Table 2:** MicroRNAs differentially expressed between Ames dwarf and Normal mice at young (6 months) and old (22 months) ages.

miRNA name	miRNA gene family	FC^1^	RPM^2^	PValue^3^	FDR^3^	miRNA name	miRNA gene family	FC^1^	RPM^2^	PValue^3^	FDR^3^
Young mice (6 months old)						Old mice (22 months old)					
**Commonly down-regulated**						**Commonly down-regulated**					
mmu-miR-212-3p	miR-132	12.16	69.74	1.10E-12	2.22E-10	mmu-miR-212-3p	miR-132	11.96	69.74	1.62E-11	2.18E-09
mmu-miR-132-5p	miR-132	9.32	10.64	4.62E-07	2.67E-05	mmu-miR-132-5p	miR-132	8.16	10.64	1.25E-05	6.29E-04
mmu-miR-132-3p	miR-132	6.87	2055.67	2.39E-09	2.41E-07	mmu-miR-132-3p	miR-132	7.38	2055.67	7.27E-10	7.34E-08
mmu-miR-21a-3p	miR-21	4.17	380.77	1.78E-07	1.44E-05	mmu-miR-21a-3p	miR-21	5.26	380.77	1.87E-09	1.51E-07
mmu-miR-21a-5p	miR-21	3.75	14917.59	2.15E-11	2.89E-09	mmu-miR-21a-5p	miR-21	4.33	14917.59	1.72E-13	3.47E-11
**Commonly up-regulated**						**Commonly up-regulated**					
mmu-miR-7a-2-3p	miR-7	17.79	24.83	3.40E-13	1.37E-10	mmu-miR-7a-2-3p	miR-7	52.52	24.83	9.07E-20	3.66E-17
mmu-miR-217-5p	miR-217	10.03	10.53	3.72E-07	2.50E-05	mmu-miR-217-5p	miR-217	49.58	10.53	5.57E-06	3.21E-04
mmu-miR-465c-5p	miR-465	8.06	30.26	6.17E-05	2.49E-03	mmu-miR-465c-5p	miR-465	7.37	30.26	2.39E-04	6.12E-03
mmu-miR-470-5p	miR-743	6.59	15.76	1.56E-03	1.86E-02	mmu-miR-470-5p	miR-743	11.99	15.76	2.42E-04	6.12E-03
mmu-miR-871-5p	miR-743	5.30	23.38	3.58E-04	9.64E-03	mmu-miR-871-5p	miR-743	8.75	23.38	2.28E-05	1.02E-03
mmu-miR-7a-5p	miR-7	3.33	56.07	4.58E-04	9.73E-03	mmu-miR-7a-5p	miR-7	3.62	56.07	1.00E-04	3.38E-03
mmu-miR-107-3p	miR-103	2.15	2359.91	7.75E-04	1.29E-02	mmu-miR-107-3p	miR-103	3.19	2359.91	5.55E-07	3.74E-05
**Down-regulated in young mice only**						**Down-regulated in old mice only**					
mmu-miR-133b-3p	miR-133	5.19	48.09	6.00E-05	2.49E-03	mmu-miR-215-5p	miR-192	8.45	191.47	5.96E-04	1.09E-02
mmu-miR-501-3p	miR-500	4.70	20.78	1.27E-03	1.70E-02	mmu-miR-142a-3p	miR-142	5.25	586.99	5.41E-05	2.18E-03
mmu-miR-133a-3p	miR-133	4.01	513.58	3.57E-04	9.64E-03	mmu-miR-296-5p	miR-296	3.02	88.46	8.98E-04	1.58E-02
mmu-miR-3102-3p.2-3p	miR-3102	3.17	51.46	2.74E-06	1.38E-04	mmu-miR-101b-3p	miR-101	2.26	1990.35	4.73E-04	1.01E-02
mmu-miR-582-5p	miR-582	3.14	16.45	1.43E-03	1.75E-02						
mmu-miR-330-3p	miR-330	2.90	26.73	5.95E-04	1.05E-02	**Up-regulated in old mice only**					
mmu-let-7a-1-3p	let-7	2.75	162.24	5.30E-04	1.03E-02	mmu-miR-370-3p	miR-370	5.43	9.62	9.03E-05	3.32E-03
mmu-miR-148b-3p	miR-148	2.54	715.73	1.27E-03	1.70E-02	mmu-miR-547-3p	miR-547	4.36	413.17	3.27E-04	7.35E-03
mmu-miR-3102-3p	miR-3102	2.38	104.61	4.30E-04	9.66E-03	mmu-miR-201-5p	miR-743	4.35	43.39	1.14E-04	3.55E-03
mmu-miR-28a-5p	miR-28	2.11	436.17	1.29E-03	1.70E-02	mmu-miR-201-3p	miR-743	3.80	40.54	5.81E-04	1.09E-02
mmu-miR-28c	miR-28	2.11	436.17	1.31E-03	1.70E-02	mmu-miR-1249-3p	miR-1249	2.42	609.50	5.35E-04	1.08E-02
mmu-miR-34a-5p	miR-34	2.04	247.19	7.98E-04	1.29E-02	mmu-miR-1247-5p	miR-1247	2.39	665.42	1.09E-03	1.84E-02
mmu-miR-28a-3p	miR-28	2.00	79.11	1.20E-03	1.70E-02	mmu-miR-652-3p	miR-652	2.18	3266.88	1.46E-04	4.21E-03
						mmu-miR-181b-5p	miR-181	2.06	7681.97	2.93E-04	6.97E-03
**Up-regulated in young mice only**											
mmu-miR-215-5p	miR-192	9.63	191.47	3.13E-04	9.64E-03						
mmu-miR-465abc-3p	miR-465	7.33	7.41	2.79E-04	9.40E-03						
mmu-miR-463-5p	miR-463	5.96	11.26	2.17E-04	7.98E-03						
mmu-miR-181a-2-3p	miR-181	2.73	2361.59	1.41E-03	1.75E-02						
mmu-miR-92a-1-5p	miR-25	2.61	32.62	4.12E-04	9.66E-03						
mmu-miR-150-5p	miR-150	2.45	9575.94	1.21E-03	1.70E-02						
mmu-miR-434-3p	miR-434	1.97	7570.42	5.37E-04	1.03E-02						
mmu-miR-128-3p	miR-128	1.93	166.76	1.63E-03	1.88E-02						
mmu-miR-92a-3p	miR-25	1.89	97651.91	4.30E-04	9.66E-03						
mmu-miR-130a-3p	miR-130	1.88	6489.13	5.94E-04	1.05E-02						

^1^Fold change between groups

^2^Average reads per million (RPM) for each miRNA for the groups being compared

^3^P Value and False discovery rate. miRNAs with FDR<0.05 were considered as differentially expressed between groups

**Fig 4:**
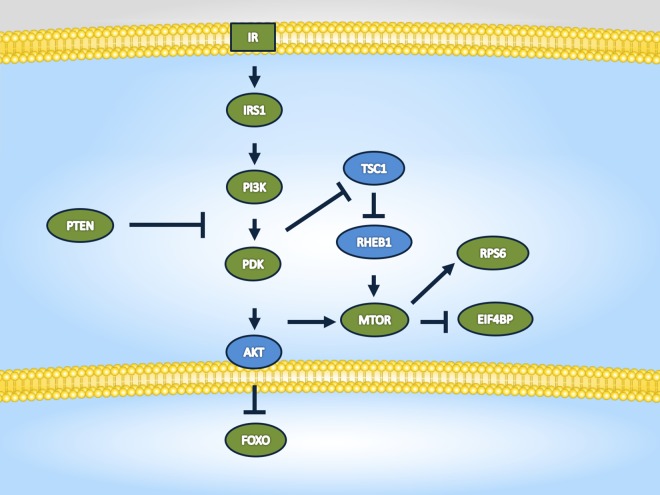
Schematic representation of the main target genes of the microRNAs differentially regulated between Normal (N) and Ames dwarf (df/df) mice at both ages (6 and 22 months). Green–target gene of a regulated miRNA; Blue–non regulated target gene.

The analysis of enriched pathways and gene ontology (GO) terms of target genes from the differentially regulated miRNAs affected by aging (young vs. old) or genotype (N vs. df/df) is presented in S2–S5 Tables. Venn diagram analysis of enriched pathways and GO terms pointed to 16 biological processes commonly regulated among all categories, including: cell differentiation, developmental maturation, anatomical structure development, cell death, growth, cell motility, cytoskeleton organization, cell division, cell morphogenesis, chromosome organization, circulatory system process, and cell cycle. Among the commonly regulated pathways between categories there was FoxO, mTOR, MAPK, PI3K-Akt, AMPK signaling pathways and Phosphatidylinositol signaling system. In addition, the enriched pathways and GO terms for the target genes of the 12 miRNAs commonly regulated between N and df/df mice at young and old ages is presented in Table 3. The main signaling pathways target by the miRNAs regulated between N and df/df mice at both young and old ages are also summarized in Fig 4. The insulin (Fig A in S1 Fig), Pi3k/Akt (Fig B in S1 Fig), FoxO (Fig C in S1 Fig) and mTOR (Fig D in S1 Fig) signaling pathways are represented in more details in the S1 Fig provided.

**Table 3:** Enriched KEGG pathways and GO Terms for biological processes for the genes targeted by miRNA differentially expressed between Ames dwarf and Normal mice at both young and older ages.

Pathways and GO Terms	P value	Genes	miRNAs
**KEGG pathways**			
Pathways in cancer	0.008	77	11
PI3K-Akt signaling pathway	0.021	66	11
MAPK signaling pathway	0.036	50	11
Regulation of actin cytoskeleton	0.024	45	11
Proteoglycans in cancer	0.002	44	11
Ras signaling pathway	0.007	42	11
Rap1 signaling pathway	0.030	42	11
cAMP signaling pathway	0.023	41	11
cGMP-PKG signaling pathway	0.023	37	11
Axon guidance	0.004	33	11
FoxO signaling pathway	0.007	32	11
AMPK signaling pathway	0.007	31	11
Signaling pathways regulating pluripotency of stem cells	0.008	31	11
Insulin signaling pathway	0.036	31	11
Adrenergic signaling in cardiomyocytes	0.036	30	11
Hippo signaling pathway	0.014	26	11
Prostate cancer	0.005	24	11
mTOR signaling pathway	2.39E-04	22	11
Melanoma	0.043	17	11
Glioma	0.007	16	11
Protein processing in endoplasmic reticulum	0.023	37	10
Glutamatergic synapse	0.008	23	10
Estrogen signaling pathway	0.021	22	10
Adherens junction	0.030	15	10
Acute myeloid leukemia	0.031	14	10
Morphine addiction	4.58E-05	26	9
GABAergic synapse	1.75E-07	20	9
Hedgehog signaling pathway	0.041	14	8
Long-term depression	0.031	14	7
Other types of O-glycan biosynthesis	0.021	7	6
**GO Terms Biological Processes**			
Anatomical structure development	1.16E-140	769	12
Embryo development	2.48E-43	220	12
Anatomical structure formation involved in morphogenesis	4.79E-27	180	12
Cellular nitrogen compound metabolic process	1.74E-15	701	11
Biosynthetic process	2.20E-10	595	11
Cell differentiation	5.68E-75	565	11
Cellular protein modification process	8.65E-12	375	11
Cellular component assembly	9.90E-06	207	11
Cell cycle	2.64E-04	171	11
Cell morphogenesis	9.09E-26	163	11
Homeostatic process	8.60E-09	161	11
Cell death	0.002	144	11
Chromosome organization	2.08E-22	134	11
Cytoskeleton organization	7.25E-07	133	11
Cell motility	8.02E-11	126	11
Cell-cell signaling	0.007	105	11
Cell division	5.37E-07	99	11
Growth	1.42E-06	86	11
Developmental maturation	1.55E-11	46	11
Circulatory system process	0.001	35	11
Regulation of neural precursor cell proliferation	0.039	9	8
Vasculogenesis involved in coronary vascular morphogenesis	0.002	7	7

## Discussion

In the current study we observed that the abundance of 22 and 33 of the 404 identified ovarian miRNAs changed with age in N and df/df mice, respectively. Of these, only three miRNAs were commonly regulated with age between N and df/df mice, indicating a very divergent profile of ovarian miRNAs as both mice genotypes age. It was previously demonstrated that the size of the primordial follicle reserve decreases by approximately 90% from the ages of 0.5 to 1.5 years in N female mice [12], and we demonstrated before that the ovarian reserve of primordial follicles in df/df mice is three times larger than in N mice [19, 20]. Therefore, our finding indicates that changes in the ovarian reserve with aging are reflected in changes in the ovarian miRNA profile of N and df/df mice. We also demonstrated that 46 different miRNAs are regulated between N and df/df mice at young and old ages, of which 23 are exclusively regulated in young mice, 12 exclusively in old mice and 12 commonly regulated at young and old ages. It can be hypothesized that differences in the expression of miRNAs between df/df and N mice and the differential impact of the aging process on their expression could be an important contributing factor for the preservation of a younger ovarian phenotype in df/df mice. Ovarian aging does not just affect the size of the ovarian follicular reserve but also affect tissue composition, including stromal fibrosis, changes in vascularization and infiltration of immune cells [39, 40]. Therefore, the changes detected in the miRNA profile with aging and between genotypes are also a reflection of changes in ovarian tissue composition. As mentioned before, miRNAs can also be secreted to the extracellular space [4] and have a role in intercellular communication in a hormone-like pattern between different cell types [5]. Consequently, independent of its source, the ovarian detected miRNAs of this study can have an overall impact on the ovarian physiology, affecting antral follicle development, oocyte quality and primordial follicular activation, which are well known hallmarks of ovarian aging [41].

The top five most expressed ovarian miRNAs detected in the current study target several pathways involved in the regulation of cell growth and apoptosis. Specifically, the FoxO, MAPK, AMPK, mTOR, PI3K/Akt and TGF-β signaling pathways were targeted significantly by these miRNAs, suggesting that abundant miRNAs in the ovary serve as endogenous suppressors of key growth and differentiation pathways. In fact, cell growth, division, differentiation and death were amongst the enriched GO terms for these miRNAs. Interestingly, FoxO, mTOR, PI3k/Akt and insulin signaling pathways were also the main targets of the miRNAs differentially expressed between N and df/df mice, suggesting that its regulation by these miRNAs may be associated to the maintenance of a younger ovarian phenotype in older df/df mice. The PI3K/Akt, FoxO and mTOR signaling pathways are essential for the maintenance of the quiescent stage and initiation of primordial follicle growth [17, 18, 29] and can be regarded as regulators of the rate of ovarian aging. In agreement with that, we have previously observed using the same ovarian RNA samples as the ones employed in the current study for RNASeq transcriptome evaluation and observed that ovarian expression of the Pi3k/Akt pathway is one of the main regulated pathways in df/df mice compared to N mice [20]. In addition, FoxO3a phosphorylation was reduced in primordial follicles of GH-deficient df/df mice [19, 42], suggesting that inhibition of this pathway (insulin/PI3k/Akt/FoxO) is essential for maintenance of primordial follicle quiescence. The PI3k, MAPK and mTOR pathways are considered survival pathways and their deregulation is frequently associated to abnormal cellular proliferation and inhibition of apoptosis [43]. Aberrant expression of genes in these pathways is observed in several types of cancers, including ovarian cancers [44], and not surprisingly these same pathways are well known for being involved in cellular senescence and aging [43].

The miR-17 family (mmu-miR-17, mmu-miR-20a and mmu-miR-93) was highly expressed within the ovaries and down-regulated with age exclusively in df/df mice. The miR-17 gene family is located at the same cluster (< 10 kb spaced on mouse chromosome 14) with mmu-miR-92a, the highest expressed ovarian miRNA in our samples, which was up-regulated in young df/df in comparison to young N mice. The miR-17 gene family is involved in the regulation of stem cell differentiation [45], and indeed signaling pathways regulating pluripotency in stem cells was one of the enriched pathways observed to be differentially regulated with aging in df/df mice. The miRNAs located in the miR-17/92 cluster are considered oncogenes and are frequently over-expressed in malignant cells, having the phosphatase and tensin homologue (PTEN) as one of their main targets [46]. Despite that, PTEN gene expression was not changed with age or between genotypes in the RNASeq experiment using the same set of samples [20]. Specifically, miR-20a overexpression is associated with increased proliferation and invasion in ovarian cancer cells [47], and cell division, motility and differentiation were among the enriched GO terms for miRNAs regulated with age in df/df mice, further suggesting this. In addition, several of the identified pathways are related to cancer development in various tissues. Both miR-17 and miR-20a also directly target TGFBRII [46], a gene well-known to be directly involved in the regulation of primordial follicle activation and ovarian aging [7]. Interestingly, miR-145, the fifth most expressed ovarian miRNA in our study, also targets TGFBRII, and is a known regulator of primordial follicle activation [7]. TGFBRII was up-regulated with age in N mice and down-regulated between old df/df and old N mice in the RNASeq experiment using the same set of samples [20], further suggesting the importance of these miRNAs in the ovarian aging.

The miR-21 family members were also among the most expressed miRNAs in the ovaries. Interestingly, these miRNAs were strongly down-regulated in ovaries of both young and older df/df mice compared to N mice. Both the -5p and -3p arms of miR-21 were downregulated to a similar extent, which suggests regulation at the level of initial transcription of the miR-21 precursor. In fact, this same pattern of regulation was observed for other seven miRNAs (mmu-miR-29c, mmu-miR-296, mmu-miR-130b, mmu-miR-17, mmu-miR-434, mmu-miR-181c, mmu-miR-132), which further confirms the validity of our analysis and suggests miRNA regulation at the gene expression level [48]. Although it is found at much lower levels, it has been suggested that the 3p arm can also possess regulatory activity [49]. miR-21 is considered an oncogene and is the most commonly over-expressed miRNA in cancerous tissues [23]. Overexpression of miR-21 was detected in ovarian epithelial carcinoma [50], and was inversely correlated with drug sensitivity and progression-free survival [51]. miR-21 is known for regulating cell growth and proliferation by targeting PTEN, and therefore its over-expression is associated with the activation of the Pi3k/Akt pathway [52]. The Pi3k/Akt signaling pathway was one of the main targets of differentially expressed miRNA between young and old N and df/df mice. In addition, among the enriched GO terms for biological processes it was observed that cell death, motility, proliferation, and differentiation are represented and are terms associated to the development of neoplasic lesions. As mentioned before, activation of the Pi3k/Akt pathway is also a key process in the initiation of primordial follicle growth [18], and PTEN knockout mice exhibit premature ovarian failure due to over-activation of the primordial follicle pool [29]. This evidence points to miR-21 as a strong candidate for regulating ovarian aging in df/df mice, through regulation of the Pi3k/Akt pathway. In agreement with this, we previously demonstrated a reduced FoxO3a phosphorylation in oocytes contained in primordial follicles of df/df mice [42], which is one of the main transcription factor targeted by the Pi3k/Akt pathway. As mentioned before, the Pi3k/Akt pathway component genes are highly regulated when evaluated by RNASeq in the ovaries of the same df/df and N mice [20]. Additionally, miR-21-5p was up-regulated in cumulus cells from oocytes of poor-responder older women undergoing IVF procedures [53], indicating that the down-regulation of miR-21-5p observed in df/df mice can be also associated to increased fertility potential.

miR-132 and -212 are both members of the miR-132 gene family and were also strongly down-regulated in young and old df/df mice in comparison to N mice. Both miRNAs are known for being up-regulated in murine granulosa cells following LH/hCG induction [54]. Therefore, our findings are not unexpected. Ames dwarf mice have an underdeveloped pituitary and decreased serum levels of gonadotropins (luteinizing and follicle stimulating hormones, LH and FSH) in comparison to N mice [55]. The miR-132/mir-212 cluster can also regulate hematopoietic stem cells survival during aging by regulating FoxO3 expression [56]. Deletion of miR-132/212 in hematopoietic cell lines increases their survival by preventing abnormalities during aging in response to nutritional stress [56]. miR-132 and -212 are important regulators of cell cycling and renewal as well as survival and autophagy [57]. Therefore, since FoxO3 is also directly involved in primordial follicle activation and maintenance of the dormancy status [17], we can expect that the down-regulation of these miRNA observed in df/df mice can be also involved with the delayed ovarian aging in df/df mice. In agreement with this, we previously observed in this same set of samples that ovarian *Foxo3a* mRNA expression was higher in old df/df than N mice, although not different between young df/df and N mice [42]

miR-434-3p was one the most expressed miRNAs in ovarian samples from the current study and was observed to be down-regulated with age in df/df mice. In addition, miR-434-3p expression was also up-regulated in young df/df compared to young N mice. A previous study has shown that miR-434 up-regulation in the skeletal muscle tissue of aged mice is prevented by calorie restriction [58]. There is very little published data on the mRNAs targeted by miR-434, but it is known for targeting a retrotransposon-like gene (RTL1) which has a role in genomic imprinting of parental origin [59], although not changed between ages or genotypes in the RNASeq evaluation [20]. Furthermore, miR-181b-5p was also among the most expressed ovarian miRNAs and its variants were up-regulated in young (mmu-miR-181a-2-3p) and aged df/df mice (mmu-miR-181b-5p). Likewise, miR-181c-5p and -3p were down-regulated with age exclusively in df/df mice. The mir-181 family has a role in differentiation of hematopoietic cells [60], and was shown to be induced by TGF-β ligands [61]. One of the main targets of miR-181 is the ATM mRNA, an important cell cycle checkpoint kinase [61] that has role in repairing double strand DNA breaks [62]. Overexpression of miR-181a in hepatocytes can suppress SIRT1 at the protein level and therefore reduce insulin sensitivity [63]. Neither, *Atm* or *Sirt1* gene expression was regulated in between ages or genotype in these same samples [20]. We observed that the insulin signaling pathway was among the main predicted enriched pathways for df/df mice with age. In astrocytes, the reduction of miR-181a expression was associated with reduced cell death, reduced oxidative stress, and increased mitochondrial function [64]. These processes can play an essential role in protecting the oocyte from environmental damage, therefore reducing potential embryonic abnormalities and preserving fertility. In the ovary, double stranded DNA breaks have been shown to increase with age and were associated with increased rate of primordial follicle loss [65].

Several members of the miR-465 and miR-743 families were up-regulated in both young and aged df/df mice, although they were expressed at lower levels than the previously discussed miRNAs. miR-465 was previously detected as one of the most highly expressed miRNAs in the newborn mouse ovary, despite being undetectable in the adult ovary [6]. The mir-465 cluster is located in the X chromosome, and is strongly expressed in the testis, although not detected in other tissues [6]. Despite the low levels detected in the ovaries of N mice at young and older ages, expression of members of this cluster was up-regulated in the ovaries of df/df mice. This finding suggests that the preservation of a high expression of this exclusively newborn miRNA could be related to the preservation of the younger ovarian phenotype previously observed in df/df mice [66]. While little is known about miR-743, one study demonstrated that miR-743 is down-regulated by increased oxidative stress, which in turn increases translation of genes involved in cellular protection mechanisms [67].

The serum from the same mice used in the current study was also submitted to miRNA sequencing and the results suggest a specific serum miRNA signature associated with healthy aging in df/df mice [28]. When we overlap these results with our current ovarian regulated miRNAs in df/df and N mice, we observed that miR-146a-5p was about 5 times more expressed in old than young N mice in both serum and ovarian samples, although its expression did not change in df/df mice in both studies [28]. In addition, serum miR-592-5p was increased in df/df mice with age but did not change in N mice [28]. We also observed an increase of miR-592-5p with age in df/df but not in N mice. Serum miRNAs have been used as a tool for diagnosis of a variety of conditions. In this sense, the serum miRNA profile of woman with ovarian epithelial cancer revealed several differentially regulated miRNAs that can be potentially used as markers for early diagnosis [68]. Serum miR-205 was higher in ovarian cancer patients and had the best diagnostic accuracy [68]. In our study miR-205-5p expression was six times higher in old than young N mice, although it did not change in df/df mice with age. This further point to an association between aging and ovarian cancer, and to miRNAs as important biomarkers of both conditions. miR-34a was also up-regulated in the serum of cancer patients [68] and we observed that it was less expressed in the ovaries of young df/df mice in comparison to N mice. Therefore, this evidence suggests that a specific serum and ovarian miRNA profile is associated with ovarian aging and cancer development, and can be involved directly in the pathogenesis of these conditions or serve as useful biomarkers for early diagnosis.

Overall, in the present study we found 54 different miRNA families differentially expressed between young and old N and df/df mice, providing the basis for understanding how aging can regulate the ovarian miRNA network. Many genes likely to be targeted by these differentially expressed miRNAs are involved in the FoxO, mTOR, Pi3k/Akt and insulin signaling pathways, which were shown to be regulated at the mRNA level evaluating the same ovaries from this mice in a RNASeq experiment [20]. These pathways are well known to be involved in maintenance of primordial follicle quiescent stage, cellular senescence and ovarian cancer development. Therefore, they are interesting targets as biomarkers for diagnosis of these conditions, as well as possible central players in the maintenance of the younger ovarian phenotype in df/df mice. However, these conclusions must be considered as tentative. Although these miRNAs have been shown to regulate specific mRNAs and its translation in several cell types, it does not necessarily mean that the same will occur in the different ovarian compartments. Thus, extensive work validating these interactions in the ovary is necessary and must be the goal of future studies.

## Supporting Information

S1 Fig **Fig A**–Schematic representation of the insulin signaling pathway and the target genes of the microRNAs differentially regulated between Normal (N) and Ames dwarf (df/df) mice at both ages (6 and 22 months). Yellow box–target gene of one down-regulated miRNA; Orange box–target gene of two or more down-regulated miRNA. **Fig B**–Schematic representation of the Pi3k/Akt signaling pathway and the target genes of the microRNAs differentially regulated between Normal (N) and Ames dwarf (df/df) mice at both ages (6 and 22 months). Yellow box–target gene of one down-regulated miRNA; Orange box–target gene of two or more down-regulated miRNA. **Fig C**–Schematic representation of the FoxO signaling pathway and the target genes of the microRNAs differentially regulated between Normal (N) and Ames dwarf (df/df) mice at both ages (6 and 22 months). Yellow box–target gene of one down-regulated miRNA; Orange box–target gene of two or more down-regulated miRNA. **Fig D**–Schematic representation of the mTOR signaling pathway and the target genes of the microRNAs differentially regulated between Normal (N) and Ames dwarf (df/df) mice at both ages (6 and 22 months). Yellow box–target gene of one down-regulated miRNA; Orange box–target gene of two or more down-regulated miRNA.
(DOC)

S1 Table Enriched KEGG pathways and GO Terms for biological process for the genes targeted by the top five most expressed miRNA in mice ovaries. (DOC)

S2 Table Enriched KEGG pathways and GO Terms for biological process for the genes targeted by the regulated miRNA during aging in Normal mice. (DOC)

S3 Table Enriched KEGG pathways and GO Terms for biological process for the genes targeted by the regulated miRNA during aging in Ames dwarf mice. (DOC)

S4 Table Enriched KEEG pathways and GO Terms for biological process for the genes targeted by miRNA differentially expressed between Ames dwarf and Normal mice at young (6 months) age. (DOC)

S5 Table Enriched KEEG pathways and GO Terms for biological process for the genes targeted by miRNA differentially expressed between Ames dwarf and Normal mice at old (22 months) age. (DOC)
