# Supplementary material for: Changes of Ovarian microRNA Profile in Long-Living Ames Dwarf Mice during Aging
Source: PLoS One. 2017 Jan 3;12(1):e0169213. doi: 10.1371/journal.pone.0169213 (PMC5207734; doi:10.1371/journal.pone.0169213)
Supplement: S1 Fig — Fig A–Schematic representation of the insulin signaling pathway and the target genes of the microRNAs differentially regulated between Normal (N) and Ames dwarf (df/df) mice at both ages (6 and 22 months). Yellow box–target gene of one down-regulated miRNA; Orange box–target gene of two or more down-regulated miRNA. Fig B–Schematic representation of the Pi3k/Akt signaling pathway and the target genes of the microRNAs differentially regulated between Normal (N) and Ames dwarf (df/df) mice at both ages (6 and 22 months). Yellow box–target gene of one down-regulated miRNA; Orange box–target gene of two or more down-regulated miRNA. Fig C–Schematic representation of the FoxO signaling pathway and the target genes of the microRNAs differentially regulated between Normal (N) and Ames dwarf (df/df) mice at both ages (6 and 22 months). Yellow box–target gene of one down-regulated miRNA; Orange box–target gene of two or more down-regulated miRNA. Fig D–Schematic representation of the mTOR signaling pathway and the target genes of the microRNAs differentially regulated between Normal (N) and Ames dwarf (df/df) mice at both ages (6 and 22 months). Yellow box–target gene of one down-regulated miRNA; Orange box–target gene of two or more down-regulated miRNA. (DOC) [file pone.0169213.s001.doc]

**Supplemental Figures**


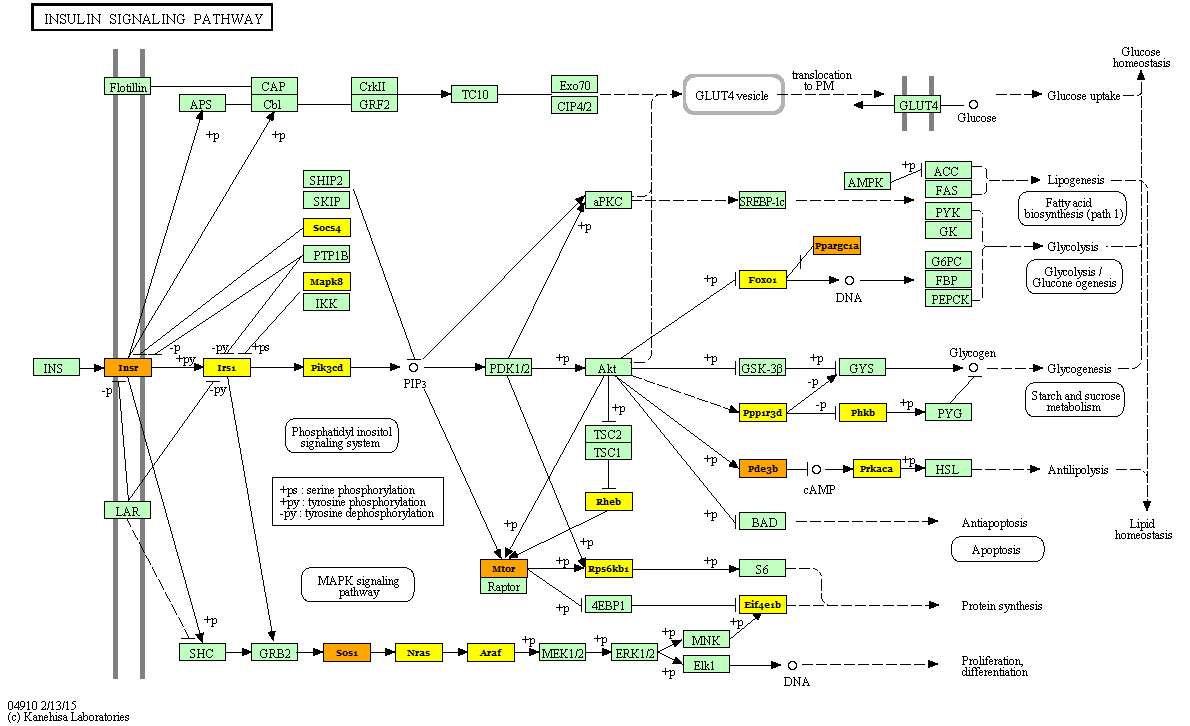


Fig A – Schematic representation of the insulin signaling pathway and the target genes of the microRNAs differentially regulated between Normal (N) and Ames dwarf (df/df) mice at both ages (6 and 22 months). Yellow box – target gene of one down-regulated miRNA; Orange box – target gene of two or more down-regulated miRNA.


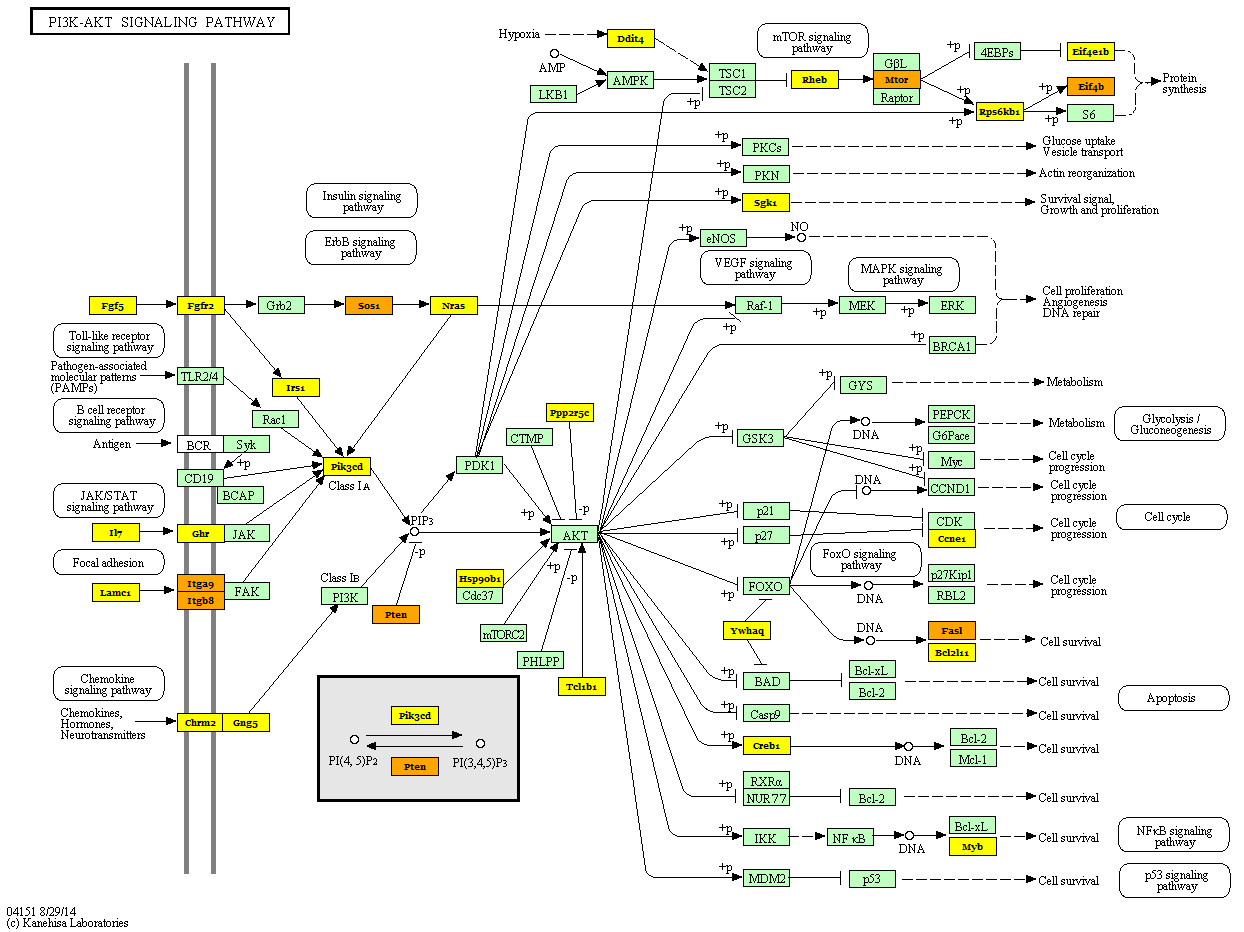


Fig B – Schematic representation of the Pi3k/Akt signaling pathway and the target genes of the microRNAs differentially regulated between Normal (N) and Ames dwarf (df/df) mice at both ages (6 and 22 months). Yellow box – target gene of one down-regulated miRNA; Orange box – target gene of two or more down-regulated miRNA.


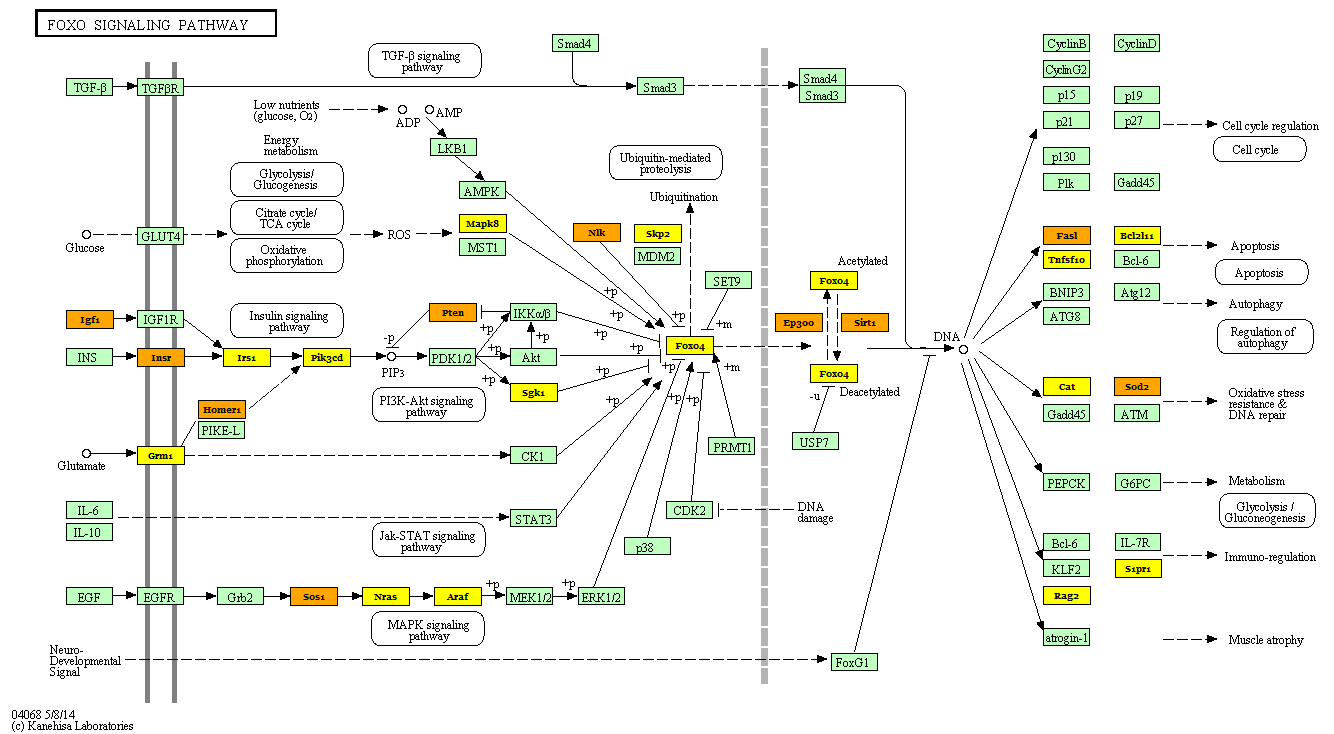


Fig C – Schematic representation of the FoxO signaling pathway and the target genes of the microRNAs differentially regulated between Normal (N) and Ames dwarf (df/df) mice at both ages (6 and 22 months). Yellow box – target gene of one down-regulated miRNA; Orange box – target gene of two or more down-regulated miRNA.


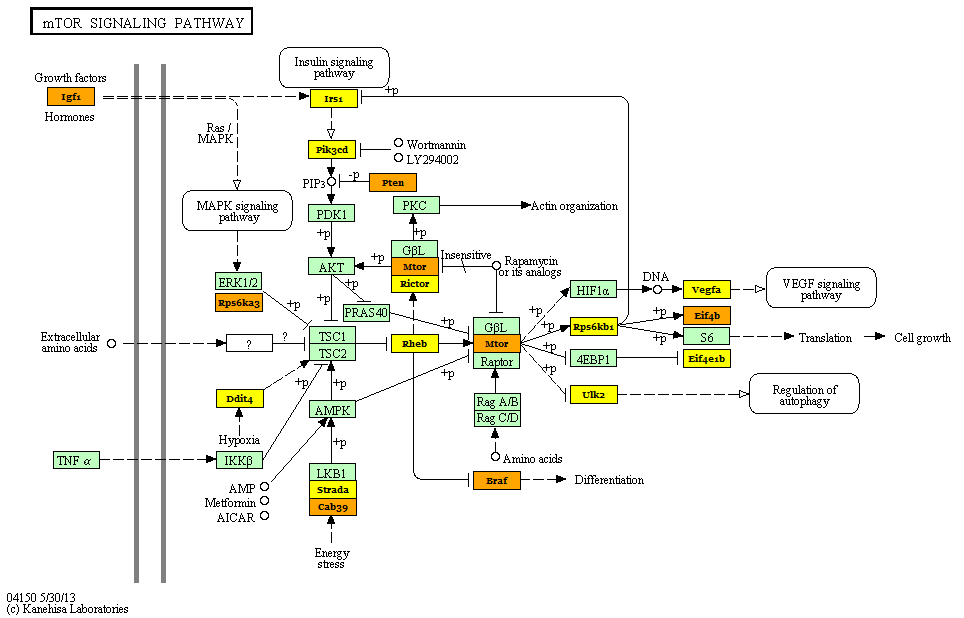


Fig D – Schematic representation of the mTOR signaling pathway and the target genes of the microRNAs differentially regulated between Normal (N) and Ames dwarf (df/df) mice at both ages (6 and 22 months). Yellow box – target gene of one down-regulated miRNA; Orange box – target gene of two or more down-regulated miRNA.
