# Supplementary material for: Changes of Ovarian microRNA Profile in Long-Living Ames Dwarf Mice during Aging
Source: PLoS One. 2017 Jan 3;12(1):e0169213. doi: 10.1371/journal.pone.0169213 (PMC5207734; doi:10.1371/journal.pone.0169213)
Supplement: S1 Table — (DOC) [file pone.0169213.s002.doc]

**Table S1 -** Enriched KEGG pathways and GO Terms for biological process for the genes targeted by the top five most expressed miRNA in mice ovaries.

| Pathways and GO Terms | P value | Genes | miRNAs |
| --- | --- | --- | --- |
| **KEGG pathways** |  |  |  |
| ECM-receptor interaction | 1.54E-10 | 15 | 5 |
| MAPK signaling pathway | 0.002 | 46 | 5 |
| Long-term potentiation | 0.004 | 19 | 5 |
| Oxytocin signaling pathway | 0.006 | 31 | 5 |
| Axon guidance | 0.01 | 29 | 5 |
| GnRH signaling pathway | 0.01 | 20 | 5 |
| Estrogen signaling pathway | 0.01 | 16 | 5 |
| Proteoglycans in cancer | 0.01 | 37 | 5 |
| Hypertrophic cardiomyopathy (HCM) | 0.01 | 19 | 5 |
| cGMP-PKG signaling pathway | 0.01 | 33 | 5 |
| mTOR signaling pathway | 0.01 | 16 | 3 |
| Amoebiasis | 0.01 | 19 | 5 |
| Phosphatidylinositol signaling system | 0.01 | 14 | 5 |
| Thyroid hormone signaling pathway | 0.01 | 25 | 5 |
| FoxO signaling pathway | 0.02 | 29 | 5 |
| Adherens junction | 0.02 | 17 | 5 |
| TGF-beta signaling pathway | 0.02 | 21 | 5 |
| Dilated cardiomyopathy | 0.02 | 18 | 5 |
| PI3K-Akt signaling pathway | 0.03 | 53 | 5 |
|  |  |  |  |
| **GO Term Biological Processes** |  |  |  |
| Anatomical structure development | 2.94E-92 | 533 | 5 |
| Cell differentiation | 1.73E-56 | 409 | 5 |
| Embryo development | 7.50E-34 | 163 | 5 |
| Cellular protein modification process | 5.05E-23 | 324 | 5 |
| Anatomical structure formation involved in morphogenesis | 1.56E-15 | 120 | 5 |
| Chromosome organization | 7.66E-12 | 87 | 5 |
| Biosynthetic process | 2.07E-09 | 434 | 5 |
| Cell morphogenesis | 3.96E-09 | 94 | 5 |
| Cellular nitrogen compound metabolic process | 5.00E-09 | 483 | 5 |
| Cytoskeleton organization | 6.86E-06 | 99 | 5 |
| Developmental maturation | 3.54E-05 | 28 | 5 |
| Cell death | 4.18E-05 | 116 | 5 |
| Growth | 0.0004 | 60 | 5 |
| Cell motility | 0.0004 | 78 | 5 |
| Circulatory system process | 0.0007 | 28 | 5 |
| Homeostatic process | 0.002 | 102 | 5 |
| Cell cycle | 0.01 | 119 | 5 |
| Cell division | 0.02 | 61 | 5 |
