# Supplementary material for: Changes of Ovarian microRNA Profile in Long-Living Ames Dwarf Mice during Aging
Source: PLoS One. 2017 Jan 3;12(1):e0169213. doi: 10.1371/journal.pone.0169213 (PMC5207734; doi:10.1371/journal.pone.0169213)
Supplement: S2 Table — (DOC) [file pone.0169213.s003.doc]

**Table S2** – Enriched KEGG pathways and GO Terms for biological process for the genes targeted by the regulated miRNA during aging in Normal mice.

| Pathways and GO Terms | P value | Genes | miRNAs |
| --- | --- | --- | --- |
| **KEGG pathways** |  |  |  |
| PI3K-Akt signaling pathway | 4.30E-09 | 104 | 18 |
| Regulation of actin cytoskeleton | 0.002 | 58 | 18 |
| Axon guidance | 2.39E-06 | 46 | 18 |
| AMPK signaling pathway | 0.004 | 34 | 18 |
| Pathways in cancer | 1.71E-07 | 104 | 17 |
| HTLV-I infection | 0.002 | 70 | 17 |
| Focal adhesion | 1.15E-05 | 63 | 17 |
| Endocytosis | 0.009 | 49 | 17 |
| Hippo signaling pathway | 0.001 | 46 | 17 |
| Signaling pathways regulating pluripotency of stem cells | 2.36E-06 | 44 | 17 |
| MAPK signaling pathway | 0.008 | 62 | 16 |
| Proteoglycans in cancer | 6.64E-07 | 62 | 16 |
| Cytokine-cytokine receptor interaction | 0.024 | 51 | 16 |
| Wnt signaling pathway | 4.20E-06 | 47 | 16 |
| FoxO signaling pathway | 4.10E-05 | 42 | 16 |
| Oxytocin signaling pathway | 0.025 | 39 | 16 |
| Insulin signaling pathway | 0.030 | 36 | 16 |
| Choline metabolism in cancer | 0.001 | 32 | 16 |
| Rap1 signaling pathway | 0.002 | 55 | 15 |
| Transcriptional misregulation in cancer | 4.10E-05 | 50 | 15 |
| Thyroid hormone signaling pathway | 2.09E-06 | 41 | 15 |
| TGF-beta signaling pathway | 3.99E-07 | 32 | 15 |
| ErbB signaling pathway | 0.006 | 25 | 15 |
| Ras signaling pathway | 0.001 | 57 | 14 |
| Neurotrophin signaling pathway | 0.015 | 32 | 14 |
| Fc gamma R-mediated phagocytosis | 0.015 | 25 | 14 |
| mTOR signaling pathway | 0.030 | 19 | 14 |
| Phosphatidylinositol signaling system | 5.18E-06 | 30 | 13 |
| T cell receptor signaling pathway | 0.027 | 28 | 13 |
| Prostate cancer | 0.011 | 27 | 13 |
| Protein digestion and absorption | 0.016 | 26 | 13 |
| Renal cell carcinoma | 0.001 | 21 | 13 |
| ECM-receptor interaction | 3.87E-28 | 29 | 12 |
| Amoebiasis | 0.001 | 28 | 12 |
| Adherens junction | 2.69E-04 | 24 | 12 |
| Glioma | 0.002 | 19 | 12 |
| Small cell lung cancer | 0.009 | 25 | 11 |
| Melanoma | 0.017 | 20 | 11 |
| Inositol phosphate metabolism | 0.004 | 19 | 11 |
| Basal cell carcinoma | 0.048 | 17 | 11 |
| Non-small cell lung cancer | 0.045 | 15 | 11 |
| Thyroid hormone synthesis | 0.004 | 14 | 11 |
| Lysine degradation | 0.025 | 12 | 11 |
| Other types of O-glycan biosynthesis | 0.035 | 10 | 9 |
| Prion diseases | 2.21E-15 | 7 | 9 |
| Hedgehog signaling pathway | 0.030 | 16 | 7 |
| Glycosaminoglycan biosynthesis - chondroitin sulfate / dermatan sulfate | 0.002 | 5 | 5 |
|  |  |  |  |
| **GO Term Biological Processes** |  |  |  |
| Anatomical structure development | 4.84E-169 | 901 | 22 |
| Biosynthetic process | 1.39E-13 | 702 | 22 |
| Cellular nitrogen compound metabolic process | 5.39E-18 | 815 | 21 |
| Cell differentiation | 6.24E-93 | 668 | 21 |
| Embryo development | 1.09E-71 | 291 | 20 |
| Anatomical structure formation involved in morphogenesis | 1.65E-37 | 221 | 20 |
| Cell morphogenesis | 1.00E-28 | 186 | 20 |
| Homeostatic process | 2.54E-08 | 179 | 20 |
| Cell death | 0.015 | 158 | 20 |
| Cellular protein modification process | 8.14E-20 | 464 | 19 |
| Cellular component assembly | 0.002 | 221 | 19 |
| Cell cycle | 4.70E-05 | 199 | 19 |
| Chromosome organization | 1.05E-22 | 148 | 19 |
| Cell motility | 2.69E-12 | 145 | 19 |
| Cell division | 1.49E-08 | 116 | 19 |
| In utero embryonic development | 0.006 | 81 | 19 |
| Circulatory system process | 0.001 | 39 | 19 |
| Cytoskeleton organization | 0.001 | 136 | 18 |
| Cell-cell signaling | 0.013 | 118 | 18 |
| Growth | 2.32E-09 | 105 | 18 |
| Developmental maturation | 9.77E-15 | 55 | 18 |
| Odontogenesis of dentin-containing tooth | 0.016 | 27 | 16 |
