# Supplementary material for: Changes of Ovarian microRNA Profile in Long-Living Ames Dwarf Mice during Aging
Source: PLoS One. 2017 Jan 3;12(1):e0169213. doi: 10.1371/journal.pone.0169213 (PMC5207734; doi:10.1371/journal.pone.0169213)
Supplement: S3 Table — (DOC) [file pone.0169213.s004.doc]

**Table S3** – Enriched KEGG pathways and GO Terms for biological process for the genes targeted by the regulated miRNA during aging in Ames dwarf mice.

| Pathways and GO Terms | P value | Genes | miRNAs |
| --- | --- | --- | --- |
| **KEGG pathways** |  |  |  |
| PI3K-Akt signaling pathway | 0.020 | 99 | 27 |
| Regulation of actin cytoskeleton | 0.001 | 75 | 27 |
| cGMP-PKG signaling pathway | 2.41E-04 | 62 | 27 |
| Pathways in cancer | 2.61E-04 | 125 | 26 |
| MAPK signaling pathway | 0.001 | 83 | 26 |
| Endocytosis | 2.82E-06 | 77 | 26 |
| Proteoglycans in cancer | 7.41E-07 | 75 | 26 |
| Axon guidance | 1.18E-11 | 69 | 26 |
| Focal adhesion | 0.007 | 66 | 26 |
| Transcriptional misregulation in cancer | 0.001 | 61 | 26 |
| Dopaminergic synapse | 0.001 | 50 | 26 |
| Oocyte meiosis | 0.032 | 41 | 26 |
| Progesterone-mediated oocyte maturation | 0.020 | 32 | 26 |
| Thyroid hormone signaling pathway | 2.44E-09 | 50 | 25 |
| AMPK signaling pathway | 2.61E-04 | 48 | 25 |
| HTLV-I infection | 0.050 | 78 | 24 |
| cAMP signaling pathway | 0.001 | 68 | 24 |
| Rap1 signaling pathway | 0.005 | 67 | 24 |
| Wnt signaling pathway | 8.18E-08 | 61 | 24 |
| Insulin signaling pathway | 0.003 | 50 | 24 |
| Adrenergic signaling in cardiomyocytes | 0.026 | 45 | 24 |
| Sphingolipid signaling pathway | 0.003 | 44 | 24 |
| GABAergic synapse | 0.003 | 28 | 24 |
| Glioma | 0.001 | 24 | 24 |
| Glutamatergic synapse | 2.41E-04 | 40 | 23 |
| Prostate cancer | 0.003 | 33 | 23 |
| Morphine addiction | 0.004 | 29 | 23 |
| Signaling pathways regulating pluripotency of stem cells | 1.12E-07 | 59 | 22 |
| Oxytocin signaling pathway | 0.004 | 56 | 22 |
| FoxO signaling pathway | 2.80E-05 | 55 | 22 |
| Neurotrophin signaling pathway | 2.61E-04 | 49 | 22 |
| T cell receptor signaling pathway | 4.75E-04 | 41 | 22 |
| ErbB signaling pathway | 0.003 | 31 | 22 |
| mTOR signaling pathway | 0.001 | 28 | 22 |
| Arrhythmogenic right ventricular cardiomyopathy (ARVC) | 0.003 | 25 | 22 |
| Hippo signaling pathway | 4.63E-10 | 60 | 21 |
| Protein processing in endoplasmic reticulum | 2.87E-04 | 59 | 21 |
| TGF-beta signaling pathway | 6.09E-05 | 37 | 21 |
| Estrogen signaling pathway | 2.61E-04 | 37 | 21 |
| Choline metabolism in cancer | 0.017 | 35 | 21 |
| Long-term depression | 0.048 | 19 | 21 |
| Dorso-ventral axis formation | 0.007 | 13 | 21 |
| Phosphatidylinositol signaling system | 0.004 | 33 | 20 |
| Long-term potentiation | 0.001 | 30 | 20 |
| Thyroid hormone synthesis | 0.003 | 20 | 20 |
| Circadian rhythm | 0.004 | 16 | 20 |
| Hepatitis B | 0.035 | 41 | 19 |
| Adherens junction | 0.003 | 32 | 19 |
| Amphetamine addiction | 2.82E-06 | 29 | 19 |
| Gap junction | 0.026 | 25 | 19 |
| Inositol phosphate metabolism | 0.009 | 23 | 19 |
| Renal cell carcinoma | 0.003 | 27 | 18 |
| Chronic myeloid leukemia | 0.036 | 26 | 18 |
| Hedgehog signaling pathway | 0.002 | 23 | 18 |
| Lysine degradation | 0.025 | 15 | 18 |
| Melanogenesis | 2.87E-04 | 40 | 17 |
| Osteoclast differentiation | 0.020 | 40 | 17 |
| Sphingolipid metabolism | 0.007 | 16 | 16 |
| Basal cell carcinoma | 0.001 | 26 | 15 |
| Notch signaling pathway | 0.032 | 19 | 15 |
| Nicotine addiction | 0.030 | 14 | 15 |
| Cocaine addiction | 4.19E-04 | 18 | 14 |
| Thyroid cancer | 0.032 | 10 | 14 |
| Fatty acid metabolism | 0.050 | 9 | 14 |
| Glycosphingolipid biosynthesis - lacto and neolacto series | 0.006 | 6 | 12 |
| Prion diseases | 3.64E-13 | 11 | 11 |
| Glycosphingolipid biosynthesis - ganglio series | 0.001 | 6 | 6 |
|  |  |  |  |
| **GO Term Biological Processes** |  |  |  |
| Cellular nitrogen compound metabolic process | 8.07E-21 | 1144 | 32 |
| Anatomical structure development | 6.08E-207 | 1216 | 31 |
| Cell differentiation | 9.66E-116 | 913 | 31 |
| Cellular protein modification process | 2.56E-32 | 682 | 31 |
| Cellular component assembly | 0.003 | 303 | 31 |
| Biosynthetic process | 2.43E-18 | 1004 | 30 |
| Embryo development | 9.36E-72 | 359 | 30 |
| Cell cycle | 1.78E-12 | 311 | 30 |
| Anatomical structure formation involved in morphogenesis | 3.26E-49 | 304 | 30 |
| Cell morphogenesis | 2.63E-48 | 278 | 30 |
| Homeostatic process | 4.44E-10 | 248 | 30 |
| Cell death | 0.001 | 228 | 30 |
| Cytoskeleton organization | 4.05E-10 | 215 | 30 |
| Cell motility | 1.01E-18 | 210 | 30 |
| Chromosome organization | 7.18E-31 | 206 | 30 |
| Growth | 5.73E-13 | 148 | 30 |
| Cell-cell signaling | 1.11E-04 | 174 | 29 |
| Cell division | 2.09E-13 | 169 | 29 |
| In utero embryonic development | 0.001 | 112 | 29 |
| Developmental maturation | 1.61E-12 | 63 | 27 |
| Circulatory system process | 0.001 | 50 | 23 |
| Odontogenesis of dentin-containing tooth | 0.032 | 34 | 22 |
