# Supplementary material for: Changes of Ovarian microRNA Profile in Long-Living Ames Dwarf Mice during Aging
Source: PLoS One. 2017 Jan 3;12(1):e0169213. doi: 10.1371/journal.pone.0169213 (PMC5207734; doi:10.1371/journal.pone.0169213)
Supplement: S4 Table — (DOC) [file pone.0169213.s005.doc]

**Table S4** – Enriched KEEG pathways and GO Terms for biological process for the genes targeted by miRNA differentially expressed between Ames dwarf and Normal mice at young (6 months) age.

| Pathways and GO terms | P value | Genes | miRNAs |
| --- | --- | --- | --- |
| **KEGG pathways** |  |  |  |
| PI3K-Akt signaling pathway | 1.56E-04 | 129 | 32 |
| Pathways in cancer | 2.18E-05 | 146 | 31 |
| cAMP signaling pathway | 3.03E-05 | 83 | 31 |
| Adrenergic signaling in cardiomyocytes | 1.26E-08 | 67 | 31 |
| cGMP-PKG signaling pathway | 0.002 | 67 | 31 |
| Insulin signaling pathway | 0.003 | 57 | 31 |
| MAPK signaling pathway | 2.67E-05 | 103 | 30 |
| Endocytosis | 7.65E-05 | 85 | 30 |
| Oxytocin signaling pathway | 0.005 | 63 | 30 |
| AMPK signaling pathway | 0.001 | 54 | 30 |
| Regulation of actin cytoskeleton | 1.12E-05 | 91 | 29 |
| Rap1 signaling pathway | 9.58E-05 | 85 | 29 |
| Ras signaling pathway | 4.68E-05 | 84 | 29 |
| Focal adhesion | 0.001 | 81 | 29 |
| Axon guidance | 1.44E-08 | 65 | 29 |
| Protein processing in endoplasmic reticulum | 0.004 | 65 | 29 |
| Choline metabolism in cancer | 0.012 | 41 | 29 |
| Proteoglycans in cancer | 1.58E-05 | 81 | 28 |
| Hippo signaling pathway | 3.03E-05 | 62 | 28 |
| Signaling pathways regulating pluripotency of stem cells | 3.03E-05 | 61 | 28 |
| Sphingolipid signaling pathway | 0.003 | 51 | 28 |
| Tight junction | 0.017 | 51 | 28 |
| Neurotrophin signaling pathway | 0.014 | 48 | 28 |
| FoxO signaling pathway | 3.20E-05 | 60 | 27 |
| Dopaminergic synapse | 0.001 | 56 | 27 |
| Wnt signaling pathway | 0.010 | 53 | 27 |
| Thyroid hormone signaling pathway | 4.82E-05 | 50 | 27 |
| TGF-beta signaling pathway | 1.21E-04 | 42 | 27 |
| Prostate cancer | 3.45E-04 | 41 | 27 |
| Estrogen signaling pathway | 0.004 | 39 | 27 |
| Renal cell carcinoma | 0.004 | 30 | 27 |
| Calcium signaling pathway | 0.016 | 63 | 26 |
| Glutamatergic synapse | 4.07E-04 | 43 | 26 |
| ErbB signaling pathway | 0.005 | 36 | 26 |
| mTOR signaling pathway | 4.24E-04 | 31 | 26 |
| Platelet activation | 0.035 | 46 | 25 |
| Dilated cardiomyopathy | 0.012 | 38 | 25 |
| Hypertrophic cardiomyopathy (HCM) | 0.014 | 36 | 25 |
| Long-term potentiation | 0.002 | 32 | 25 |
| T cell receptor signaling pathway | 0.030 | 39 | 24 |
| Morphine addiction | 0.004 | 37 | 24 |
| Adherens junction | 3.27E-04 | 35 | 24 |
| GnRH signaling pathway | 0.019 | 35 | 24 |
| Gap junction | 0.008 | 29 | 24 |
| Melanoma | 0.030 | 29 | 24 |
| Pancreatic cancer | 0.049 | 27 | 24 |
| GABAergic synapse | 1.81E-08 | 36 | 23 |
| Arrhythmogenic right ventricular cardiomyopathy (ARVC) | 1.92E-05 | 36 | 23 |
| Fc gamma R-mediated phagocytosis | 0.029 | 34 | 23 |
| ECM-receptor interaction | 3.66E-08 | 31 | 23 |
| Glioma | 0.003 | 26 | 23 |
| Colorectal cancer | 0.035 | 26 | 23 |
| Phosphatidylinositol signaling system | 0.014 | 29 | 22 |
| Dorso-ventral axis formation | 0.004 | 15 | 22 |
| Melanogenesis | 0.001 | 42 | 21 |
| Amphetamine addiction | 4.31E-05 | 29 | 21 |
| Long-term depression | 0.005 | 24 | 20 |
| Notch signaling pathway | 0.043 | 21 | 20 |
| Hedgehog signaling pathway | 0.014 | 23 | 19 |
| Bacterial invasion of epithelial cells | 0.038 | 28 | 18 |
| Basal cell carcinoma | 0.018 | 25 | 18 |
| Cocaine addiction | 0.008 | 17 | 18 |
| Thyroid cancer | 0.014 | 13 | 17 |
| Nicotine addiction | 0.014 | 17 | 16 |
| Other types of O-glycan biosynthesis | 0.013 | 13 | 16 |
| Prion diseases | 7.36E-11 | 12 | 14 |
| Glycosaminoglycan biosynthesis - heparan sulfate / heparin | 0.035 | 8 | 8 |
|  |  |  |  |
| **GO Term Biological Processes** |  |  |  |
| Anatomical structure development | 1.94E-258 | 1392 | 35 |
| Cell differentiation | 1.09E-135 | 1023 | 34 |
| Embryo development | 6.10E-89 | 411 | 34 |
| Anatomical structure formation involved in morphogenesis | 1.67E-46 | 318 | 34 |
| Cell death | 5.64E-05 | 256 | 34 |
| Cellular nitrogen compound metabolic process | 1.55E-24 | 1274 | 33 |
| Biosynthetic process | 1.07E-22 | 1125 | 33 |
| Cellular protein modification process | 1.14E-27 | 719 | 33 |
| Cellular component assembly | 2.26E-05 | 349 | 33 |
| Cell-cell signaling | 3.12E-05 | 192 | 33 |
| Cell cycle | 1.55E-07 | 312 | 32 |
| Cell morphogenesis | 1.63E-45 | 290 | 32 |
| Homeostatic process | 1.21E-11 | 276 | 32 |
| Cytoskeleton organization | 1.21E-11 | 239 | 32 |
| Chromosome organization | 1.16E-37 | 234 | 32 |
| Growth | 5.47E-14 | 162 | 32 |
| Cell motility | 6.37E-20 | 229 | 31 |
| Developmental maturation | 1.48E-18 | 77 | 31 |
| Cell division | 1.10E-11 | 176 | 30 |
| In utero embryonic development | 0.002 | 121 | 30 |
| Circulatory system process | 4.73E-07 | 62 | 28 |
| Vasculogenesis involved in coronary vascular morphogenesis | 0.002 | 9 | 16 |
