# Supplementary material for: Changes of Ovarian microRNA Profile in Long-Living Ames Dwarf Mice during Aging
Source: PLoS One. 2017 Jan 3;12(1):e0169213. doi: 10.1371/journal.pone.0169213 (PMC5207734; doi:10.1371/journal.pone.0169213)
Supplement: S5 Table — (DOC) [file pone.0169213.s006.doc]

**Table S5** – Enriched KEEG pathways and GO Terms for biological process for the genes targeted by miRNA differentially expressed between Ames dwarf and Normal mice at old (22 months) age.

| Pathways and GO Terms | P value | Genes | miRNAs |
| --- | --- | --- | --- |
| **KEGG pathway** |  |  |  |
| Endocytosis | 0.028 | 53 | 20 |
| Signaling pathways regulating pluripotency of stem cells | 0.016 | 39 | 20 |
| Hippo signaling pathway | 0.012 | 36 | 20 |
| Pathways in cancer | 0.004 | 99 | 19 |
| PI3K-Akt signaling pathway | 0.011 | 86 | 19 |
| MAPK signaling pathway | 0.008 | 70 | 19 |
| Proteoglycans in cancer | 4.12E-04 | 61 | 19 |
| Rap1 signaling pathway | 0.003 | 59 | 19 |
| cGMP-PKG signaling pathway | 0.003 | 51 | 19 |
| Axon guidance | 3.56E-05 | 46 | 19 |
| AMPK signaling pathway | 0.027 | 36 | 19 |
| Regulation of actin cytoskeleton | 0.014 | 58 | 18 |
| Ras signaling pathway | 0.003 | 56 | 18 |
| Oxytocin signaling pathway | 0.016 | 46 | 18 |
| Calcium signaling pathway | 0.040 | 45 | 18 |
| FoxO signaling pathway | 0.020 | 38 | 18 |
| Choline metabolism in cancer | 0.014 | 30 | 18 |
| ErbB signaling pathway | 0.044 | 27 | 18 |
| cAMP signaling pathway | 0.008 | 55 | 17 |
| Protein processing in endoplasmic reticulum | 0.022 | 46 | 17 |
| Adrenergic signaling in cardiomyocytes | 0.012 | 41 | 17 |
| Thyroid hormone signaling pathway | 0.001 | 37 | 17 |
| Glutamatergic synapse | 0.001 | 35 | 17 |
| Sphingolipid signaling pathway | 0.034 | 34 | 17 |
| mTOR signaling pathway | 1.09E-04 | 27 | 17 |
| Prostate cancer | 0.015 | 27 | 17 |
| Gap junction | 0.025 | 22 | 17 |
| Cholinergic synapse | 0.008 | 35 | 16 |
| Morphine addiction | 3.56E-05 | 31 | 16 |
| Estrogen signaling pathway | 0.001 | 30 | 16 |
| Phosphatidylinositol signaling system | 0.001 | 25 | 16 |
| Long-term potentiation | 0.003 | 25 | 16 |
| Glioma | 0.002 | 21 | 16 |
| Pancreatic cancer | 0.040 | 18 | 16 |
| Dopaminergic synapse | 0.035 | 39 | 15 |
| Vascular smooth muscle contraction | 0.031 | 34 | 15 |
| GABAergic synapse | 3.56E-05 | 24 | 15 |
| Arrhythmogenic right ventricular cardiomyopathy (ARVC) | 0.014 | 23 | 15 |
| Adherens junction | 0.010 | 21 | 15 |
| Melanogenesis | 0.010 | 30 | 14 |
| Long-term depression | 0.016 | 19 | 14 |
| Acute myeloid leukemia | 0.044 | 17 | 14 |
| Thyroid hormone synthesis | 0.031 | 16 | 14 |
| Amphetamine addiction | 0.003 | 20 | 12 |
|  |  |  |  |
| **GO Term Biological Processes** |  |  |  |
| Cellular nitrogen compound metabolic process | 3.40E-18 | 946 | 23 |
| Biosynthetic process | 6.74E-12 | 803 | 23 |
| Anatomical structure development | 1.41E-175 | 1016 | 22 |
| Embryo development | 8.02E-56 | 292 | 22 |
| Cell differentiation | 1.53E-92 | 748 | 21 |
| Cellular protein modification process | 1.85E-18 | 526 | 21 |
| Anatomical structure formation involved in morphogenesis | 1.57E-33 | 237 | 21 |
| Homeostatic process | 3.06E-11 | 217 | 21 |
| Cell morphogenesis | 7.24E-31 | 212 | 21 |
| Chromosome organization | 2.80E-29 | 179 | 21 |
| Growth | 1.25E-08 | 116 | 21 |
| Cellular component assembly | 1.72E-05 | 270 | 20 |
| Cell cycle | 7.59E-06 | 234 | 20 |
| Cell death | 0.013 | 184 | 20 |
| Cell motility | 1.04E-11 | 162 | 20 |
| Developmental maturation | 3.51E-12 | 56 | 20 |
| Cytoskeleton organization | 8.99E-08 | 175 | 19 |
| Cell division | 5.16E-09 | 133 | 19 |
| Circulatory system process | 3.84E-04 | 44 | 18 |
| Cytoskeleton-dependent intracellular transport | 0.016 | 30 | 17 |
| Vasculogenesis involved in coronary vascular morphogenesis | 0.016 | 7 | 8 |
